# Downregulation of protein disulfide isomerase in sepsis and its role in tumor necrosis factor-alpha release

**DOI:** 10.1186/cc6977

**Published:** 2008-08-04

**Authors:** Mian Zhou, Asha Jacob, Natalie Ho, Michael Miksa, Rongqian Wu, Subir R Maitra, Ping Wang

**Affiliations:** 1 The Feinstein Institute for Medical Research, North Shore University Hospital and Long Island Jewish Medical Center, 350 Community Drive, Manhasset, NY 11030, USA; 2 Department of Surgery, North Shore University Hospital and Long Island Jewish Medical Center, 300 Community Drive, Manhasset, NY 11030, USA

## Abstract

**Introduction:**

Protein disulfide isomerase (*PDI*) is an important factor for the protein modification step in the post-translational event. *PDI *plays an essential role in cell survival under various stress conditions. It has been reported that *PDI *can serve as a negative regulator of nuclear factor-kappa-B (*NF-κB*) and that it can inhibit lipopolysaccharide (LPS)-induced proinflammatory cytokine production in macrophages. Thus, *PDI *may be an intracellular anti-inflammatory molecule. Although we have previously shown that Kupffer cell-derived proinflammatory cytokines cause liver injury in sepsis, the effect of sepsis on *PDI *expression as well as the effect of *PDI *inhibition on cytokine production have not been investigated. We therefore hypothesized that sepsis downregulates *PDI *expression and that the inhibition of *PDI *promotes proinflammatory cytokine production.

**Method:**

Adult male rats were subjected to sepsis by cecal ligation and puncture (CLP) or endotoxemia (continuous infusion of 1 μg/kg body weight LPS by an osmotic pump) for 20 hours. Hepatic tissues were collected and *PDI *gene expression was determined. In additional experiments, cells from a macrophage-like cell line, RAW 264.7, were treated with 100 ng/mL LPS for 4 hours and protein expressions were measured. RAW 264.7 cells were also treated with bacitracin, a specific *PDI *inhibitor, for 24 hours, and tumor necrosis factor-alpha (*TNF-α*) gene and protein expression as well as its release in the cell supernatant were determined. To further confirm the beneficial effect of *PDI *in sepsis, RAW 264.7 cells were transfected with *PDI *short interfering RNA (siRNA) and *PDI *gene expression and *TNF-α *release were measured by quantitative polymerase chain reaction and enzyme-linked immunosorbent assay, respectively.

**Results:**

*PDI *gene expression was significantly decreased by 28% and 69% at 20 hours after CLP or LPS infusion, respectively. LPS also decreased *PDI *protein expression by 33% in RAW 264.7 cells. Incubation of RAW 264.7 cells with bacitracin significantly increased *TNF-α *gene expression and *TNF-α *release as well as its cellular levels in a dose-dependent manner. Transfection of RAW 264.7 cells with *PDI *siRNA produced an average 36.8% inhibition of the *PDI *gene expression. This downregulation was correlated with a 3.19-fold increase in *TNF-α *release into the cell supernatant.

**Conclusion:**

Taken together, these results suggest that downregulation of *PDI *by sepsis significantly increases proinflammatory cytokine production. Thus, prevention of *PDI *downregulation in sepsis may be a novel approach to attenuate hyperinflammation and to reduce tissue injury under such conditions.

## Introduction

Infection and sepsis continue to be the most common causes of death in noncardiac intensive care units [1-4]. Evidence indicates that, in the US alone, more than 750,000 patients develop sepsis and septic shock each year with an overall mortality of 28.6% [5]. Severe sepsis is a common, expensive, and frequently fatal condition with as many deaths annually as those from acute myocardial infarction. The sepsis model of cecal ligation and puncture (CLP) mimics many features of clinical sepsis-peritonitis [6-14]. By using the CLP model of sepsis in the rat, we have shown that organ dysfunction occurred early after sepsis [14-18] and that the liver residential macrophages, Kupffer cells, play an important role in producing proinflammatory cytokines (for example, tumor necrosis factor-alpha [*TNF-α*]) in sepsis [19,20]. It is encouraging, however, that the complex pathophysiology of sepsis is becoming better understood as more studies are being reported. These studies are shedding light on the fundamental mechanisms of the pathogenesis of sepsis and are providing novel therapeutic approaches to modulate various pathological processes under such conditions.

Protein disulfide isomerase (*PDI*) catalyses the formation, breakage, and rearrangement of disulfide bonds within a molecule. This catalysis is an important post-translational event in the biosynthesis of many extracellular proteins that are usually coupled to the process of protein folding [21]. Disulfide formation involves the endogenous oxidized and reduced forms of glutathione and is catalysed by *PDI *in the endoplasmic reticulum[22]. The highly oxidative environment of the endoplasmic reticulum directs the catalytic action of the *PDI*-related proteins mainly toward the formation of disulfide bonds of proteins [23,24]. Among various tissues, the liver contains the largest amount of *PDI *protein, followed by the kidneys and fat tissues, and it has been shown that fasting and refeeding affect the *PDI *protein and its enzyme activities [25]. *PDI *is one of the endoplasmic reticulum stress proteins and it plays an essential role in cell survival under stress conditions [26]. These proteins also have other properties, such as proteolytic activities and the capacity of binding calcium, ATP, or other small ligands [26].

Previous studies have demonstrated that proinflammatory cytokines play a critical role in the initiation and progression of sepsis syndrome and that *TNF-α*, interleukin (*IL)-1β*, and *IL-6 *are important mediators of hemodynamic, metabolic, and immunologic alterations in the host during sepsis [27-31]. In this regard, it has been reported that *PDI *is a negative regulator of nuclear factor-kappa-B (*NF-κB*) and can inhibit cytokine production in macrophages after lipopolysaccharide (LPS) stimulation, suggesting that *PDI *may serve as an intracellular anti-inflammatory molecule [32]. Although *PDI *has been implicated in tumor- or apoptosis-associated conditions [33,34], its role in sepsis has not been investigated. In the present study, we determined PDI gene expression in the liver during sepsis and endotoxemia. Because previous studies have shown that Kupffer cell-derived proinflammatory cytokines play a major role in sepsis-induced liver injury [19,20], we also investigated the expression of *PDI *in cells of the macrophage-like cell line, RAW 264.7, after incubation with LPS. In addition, the specific PDI inhibitor, bacitracin, was used to determine the effect of *PDI *inhibition on *TNF-α *gene expression and production in the RAW 264.7 cells.

## Materials and methods

### Experimental model of sepsis

Polymicrobial sepsis was induced in adult male rats by CLP as we have previously described [35-37]. Briefly, male Sprague-Dawley rats (275 to 325 g; Charles River Laboratories, Wilmington, MA, USA) were housed in a temperature-controlled room on a 12-hour light/dark cycle and fed on a standard Purina rat chow diet (Nestlé Purina PetCare Company, St. Louis, MO, USA). Prior to the experiment, rats were fasted overnight but were allowed water *ad libitum*. The animals were anesthetized by isoflurane inhalation and a 2-cm ventral midline abdominal incision was made. The cecum was then exposed, ligated just distal to the ileocecal valve to avoid intestinal obstruction, punctured twice with an 18-gauge needle, and returned to the abdominal cavity. The incision was closed in layers and the animals were resuscitated by 3 mL/100 g body weight (BW) normal saline subcutaneously immediately after CLP to provide fluid resuscitation. Sham-operated animals underwent the same surgical procedure with the exception that the cecum was neither ligated nor punctured. Hepatic tissues were then harvested at 5 hours (early sepsis) and 20 hours (late sepsis) after CLP or sham operation for further analysis. This project was approved by the Animal Care and Use Committee of the Feinstein Institute for Medical Research (Manhasset, NY, USA).

### Administration of lipopolysaccharides

Male rats were fasted overnight but were allowed water *ad libitum*. The animals were anesthetized with isoflurane inhalation and a 1-cm ventral midline abdominal incision was made. A 200-μL mini-osmotic pump (Model 2ML1; Durect Corporation, Cupertino, CA, USA) was prefilled with LPS (*Escherichia coli *O55:B5; Sigma-Aldrich, St. Louis, MO, USA) solution (2 μg/mL in saline) and connected to a silastic catheter. The prefilled pump was primed in sterile normal saline for 2 hours at 37°C. The primed osmotic pump was then implanted subcutaneously in the rat and the silastic catheter was inserted into the abdominal cavity for the continuous infusion of LPS at a rate of 8 μL/hour for 20 hours (total dose: 1 μg/kg BW). Following the closure of the incision, the animals received 3 mL/100 g BW normal saline subcutaneously. Control animals underwent the same surgical procedure except that normal saline was infused. Hepatic tissues were collected at 20 hours after the infusion for further analysis.

### Cell culture and tumor necrosis factor-alpha measurement

Cells of the murine macrophage-like cell line, RAW 264.7, were obtained from the American Type Culture Collection (Manassas, VA, USA) and cultured in Dulbecco's modified Eagle's medium containing 10% heat-inactivated fetal bovine serum, supplemented with 15 mM HEPES (pH 7.4), 2 mM L-glutamine, 100 U/mL penicillin, and 100 μg/mL streptomycin, and placed in an incubator at 37°C in 5% CO_2_/95% air. Cells were incubated for 4 hours with LPS (100 ng/mL) and *PDI *gene expression was determined by reverse transcription-polymerase chain reaction (RT-PCR), as described below. In addition, RAW 264.7 cells were incubated for 24 hours with bacitracin, a specific *PDI *inhibitor (Sigma-Aldrich, 0.25, 1.25, and 3.75 mM) and the supernatant and cell lysate were collected for the measurement of *TNF-α*. The levels of *TNF-α *were determined by using commercially obtained enzyme-linked immunosorbent assay (ELISA) kits specific for rat *TNF-α *(BioSource International, Camarillo, CA, USA). The assay was carried out according to the instructions provided by the manufacturer.

### Assessment of protein disulfide isomerase and tumor necrosis factor-alpha gene expression

Hepatic tissues harvested from animal experiments or cells from the *in vitro *studies were fixed in RNAlate solution (Ambion, Inc., Austin, TX, USA). Total RNA was extracted using TRIzol reagent (Invitrogen, Carlsbad, CA, USA) and 4 μg RNA from hepatic tissues was reverse-transcribed to cDNA. The resulting cDNAs were amplified by PCR using specific primers for rat *PDI *(forward CTA CGA TGG CAA ATT GAG CA and reverse CTT CCA CCT CAT TGG CTG TT) and rat glyceraldehyde 3-phosphate dehydrogenase (*G3PDH*) (forward TTG TAA CCA ACT GGG ACG ATA TGG and reverse GAT CTT GAT CTT CAT GGT GCT AGG). For *TNF-α *gene expression, 1.8 μg RNA from RAW 264.7 cells was reverse-transcribed to cDNA and amplified by PCR using specific primers for mouse *TNF-α *(forward TTC TGT CCC TTT CAC TCA CTG G and reverse TTG GTG GTT TGC TAC GAC GTG G) and mouse β-actin (forward GTG GGC CGC TCT AGG CAC CAA and reverse CTC TTT GAT GTC ACG CAC GAT TTC). For both *PDI *and the *TNF-α *gene expression, the PCR was conducted at 30 cycles, each cycle consisting of 30 seconds at 94°C, 30 seconds at 60°C, and 1 minute at 72°C. Following the RT-PCR procedure, the reaction products were electrophoresed on 1.6% TBE (Tris borate-ethylenediaminetetraacetic acid)-agarose gel containing 0.22 μg/mL ethidium bromide. The gel was then photographed and the band density was analyzed by a digital image system.

### Transfection of RAW 264.7 cells with protein disulfide isomerase short interfering RNA

Silencer select predesigned *PDI *specific short interfering RNA (siRNA) (catalog number 4390771) previously annealed was obtained from Ambion, Inc., Austin, TX, USA. RAW 264.7 cells were plated at 5 × 10^5 ^cells in 12-well dishes and incubated overnight at 37°C and 5% CO_2_. Cells were then transfected with 100 nM *PDI *siRNA or negative control siRNA using Dharmafect Reagent 4 (Dharmacon RNAi Technologies, Chicago, IL, USA) in 1 mL media containing 10% serum according to the manufacturer's instructions. The transfected cells were incubated at 37°C for 48 hours. Afterward, cells were harvested for RNA isolation and the supernatant was collected for cytokine measurement. Total RNA isolated was reverse-transcribed to cDNA and used in real-time PCR with relative quantification analysis using primers specific for mouse *PDI*: forward 5'-TACCTGCTGGTGGAGTTCTATGC-3' and reverse 5'-TCGGGAGCCAGAGCTTTG-3'. The mouse β-actin primers were used as a control to quantitate the fold change in *PDI *gene expression. The supernatant collected from the transfected cells was used to measure *TNF-α *levels using ELISA kits specific for mouse *TNF-α*. The GPDH siRNA (100 nM) was used as a positive control for the transfection studies.

### Statistical analysis

All data were expressed as mean ± standard error and compared by one-way analysis of variance and Tukey's test or Student *t *test. Differences in value were considered significant if the *P *value was less than 0.05.

## Results

### Protein disulfide isomerase gene expression in the liver after cecal ligation and puncture and in RAW 264.7 after lipopolysaccharide incubation

**Figure 1:**
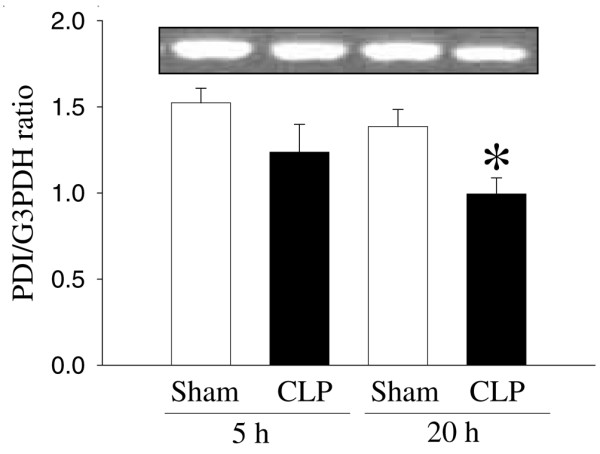
Alterations in the protein disulfide isomerase (*PDI*) gene expression in hepatic tissues at 5 and 20 hours after cecal ligation and puncture (CLP). The ratio of *PDI *and the housekeeping gene glyceraldehyde 3-phosphate dehydrogenase (*G3PDH*) is calculated. Values (n = 4 to 5/group) are presented as mean ± standard error and are compared by one-way analysis of variance and Tukey's test: *P < 0.05 versus respective sham-operated animals.

**Figure 2:**
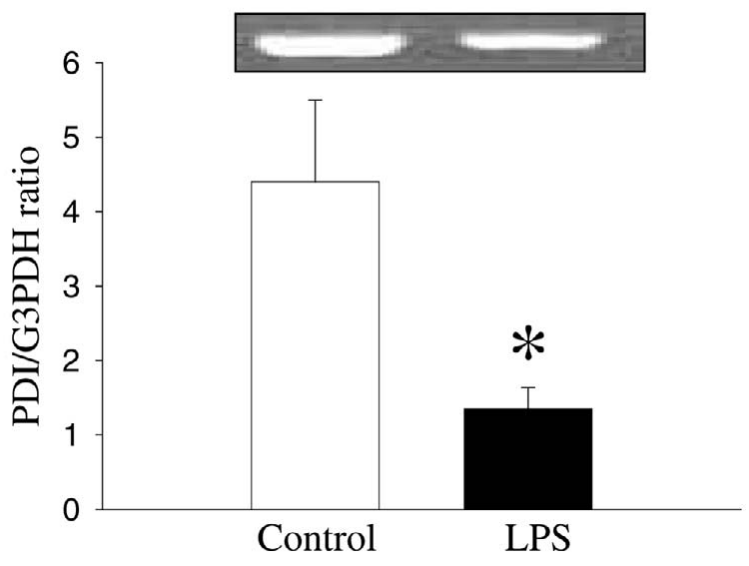
Alterations in the protein disulfide isomerase (*PDI*) gene expression in hepatic tissues after continuous infusion of lipopolysaccharide (LPS) or normal saline (control). The ratio of *PDI *and the housekeeping gene glyceraldehyde 3-phosphate dehydrogenase (*G3PDH*) is calculated. Values (n = 4 to 6/group) are presented as mean ± standard error and are compared by Student *t *test: *P < 0.05 versus control.

**Figure 3:**
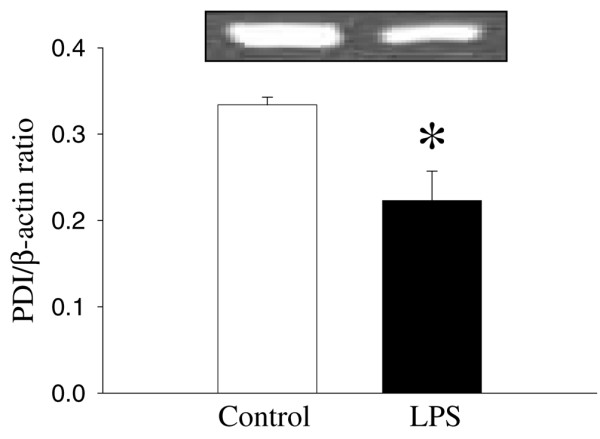
Alterations in the protein disulfide isomerase (*PDI*) protein expression in RAW 264.7 cells after stimulation of lipopolysaccharide (LPS) (100 ng/mL) for 4 hours. The ratio of *PDI *and the housekeeping gene β-actin is calculated. Values (n = 4/group) are presented as mean ± standard error and are compared by Student *t *test: *P < 0.05 versus control.

As shown in Figure 1, despite the fact that the *PDI *gene expression in hepatic tissues decreased by 19% at 5 hours after CLP, such a decrease was not statistically significant. In contrast, hepatic *PDI *gene expression decreased by 28% at 20 hours after CLP (*P *< 0.05, Figure 1). At 20 hours after the continuous infusion of LPS (1 μg/kg BW) in normal rats, the hepatic *PDI *gene expression markedly decreased by 69% (*P *< 0.05, Figure 2). This suggests that LPS may be responsible for the downregulation of the *PDI *gene expression observed 20 hours after the onset of sepsis. In cells of the cultured macrophage-like cell line, RAW 264.7, the *PDI *protein expression was also significantly reduced (by 33%) after incubation with LPS (100 ng/mL) for 4 hours (Figure 3).

### Effects of protein disulfide isomerase inhibition on tumor necrosis factor-alpha gene expression and production in RAW 264.7 cells

**Figure 4:**
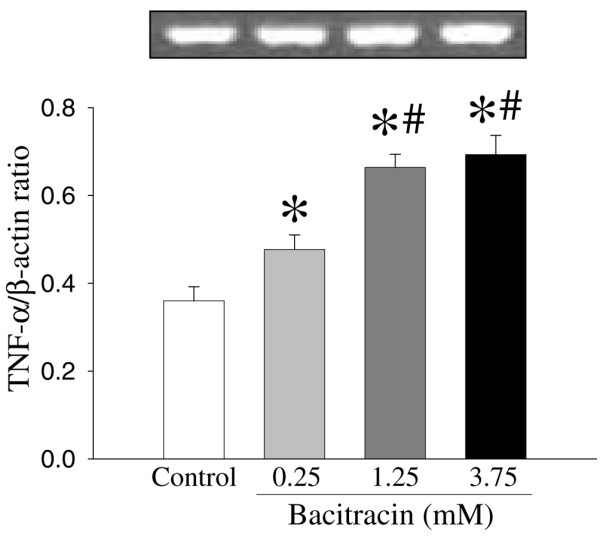
Alterations in tumor necrosis factor-alpha (*TNF-α*) gene expression in RAW 264.7 cells after culture with bacitracin (0.25, 1.25, and 3.75 mM) for 24 hours. The ratio of *TNF-α *and the housekeeping gene β-actin is calculated. Values (n = 4 to 5/group) are presented as mean ± standard error and are compared by one-way analysis of variance and Tukey's test: *P < 0.05 versus control; ^#^*P *< 0.05 versus 0.25 mM bacitracin.

**Figure 5:**
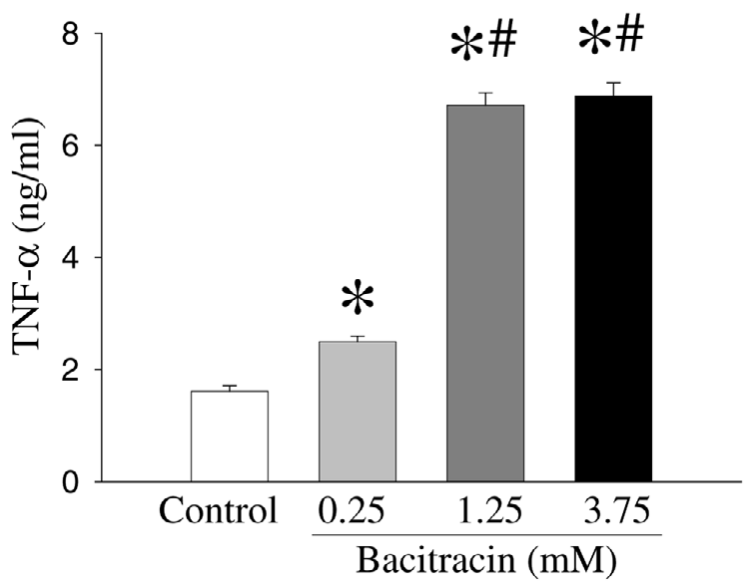
Alterations in supernatant tumor necrosis factor-alpha (*TNF-α*) levels in RAW 264.7 cells after culture with bacitracin (0.25, 1.25, and 3.75 mM) for 24 hours. *TNF-α *levels were determined by enzyme-linked immunosorbent assay. Values (n = 7 to 8/group) are presented as mean ± standard error and are compared by one-way analysis of variance and Tukey's test: *P < 0.05 versus control; ^#^*P *< 0.05 versus 0.25 mM bacitracin.

**Figure 6:**
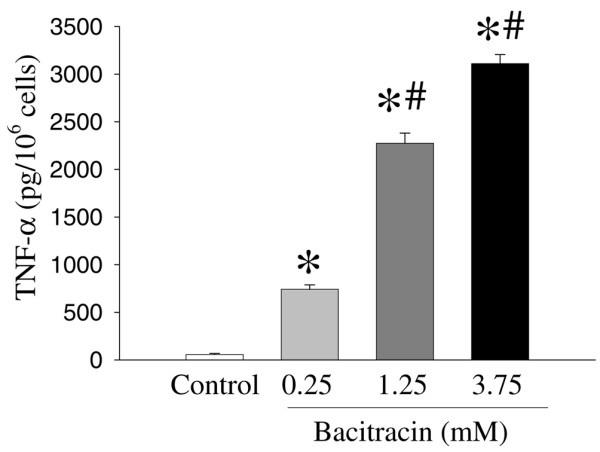
Alterations in cellular tumor necrosis factor-alpha (*TNF-α*) levels in RAW 264.7 cells cultured with bacitracin (0.25, 1.25, and 3.75 mM) for 24 hours. *TNF-α *levels were determined by enzyme-linked immunosorbent assay. Values (n = 7 to 8/group) are presented as mean ± standard error and are compared by one-way analysis of variance and Tukey's test: *P < 0.05 versus control; ^#^*P *< 0.05 versus 0.25 mM bacitracin.

To investigate the role of *PDI *in the regulation of proinflammatory cytokine *TNF-α*, we incubated RAW 264.7 cells with a specific *PDI *inhibitor, bacitracin (24-hour culture). Figure 4 shows the effect of bacitracin on the *TNF-α *gene expression in RAW 264.7 cells. Bacitracin significantly increased *TNF-α *gene expression in a dose-dependent manner. The *TNF-α *gene expression was increased by 33%, 84%, and 93% at 0.25, 1.25, and 3.75 mM bacitracin, respectively (Figure 4). Alterations in the supernatant and cellular *TNF-α *levels in cells cultured with bacitracin are shown in Figures 5 and 6. As shown in Figure 5, the supernatant levels of *TNF-α *significantly increased (by 55%) at 0.25 mM bacitracin and further increased by 317% and 327% at the higher concentrations, 1.25 and 3.75 mM, respectively (Figure 5). Similarly, cellular concentrations of *TNF-α *were markedly elevated by bacitracin in the range of 12- to 54-fold in a dose-response fashion (Figure 6).

### Effect of protein disulfide isomerase inhibition by short interfering RNA on tumor necrosis factor-alpha gene expression and release in RAW 264.7 cells

**Figure 7:**
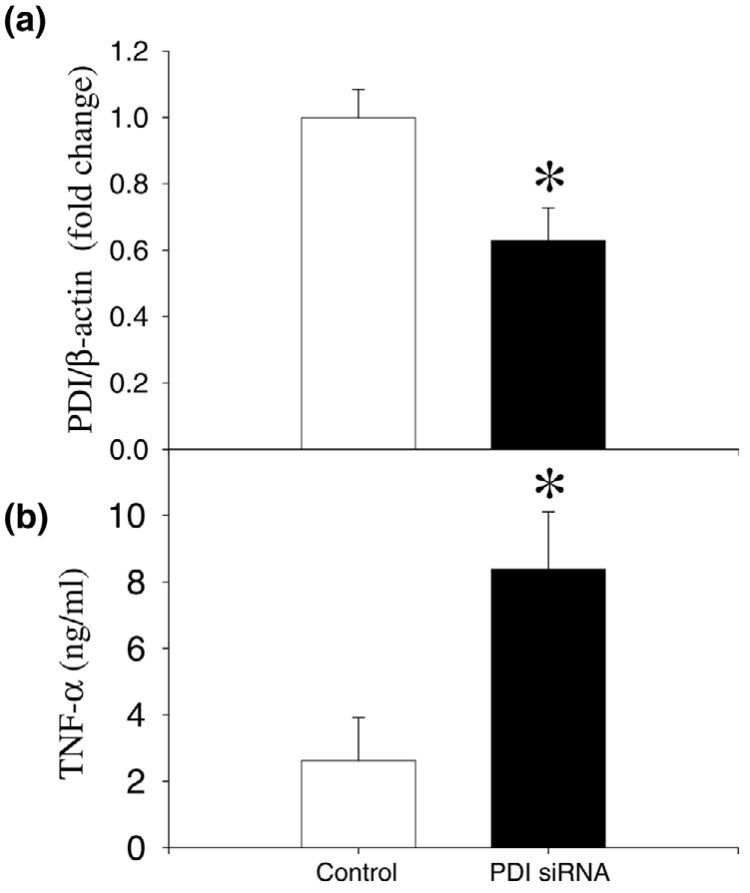
Alterations in the protein disulfide isomerase (*PDI*) gene expression and supernatant tumor necrosis factor-alpha (*TNF-α*) levels in RAW 264.7 cells transfected with *PDI *short interfering RNA (siRNA) for 48 hours. **(a) ***PDI *gene expression was determined by real-time polymerase chain reaction using specific *PDI *primers. **(b) **The *TNF-α *release into the cell supernatant was measured by enzyme-linked immunosorbent assay. Values (n = 3 to 6/group) are presented as mean ± standard error and are compared by paired Student *t *test. *P < 0.05 versus control.

To further confirm the role of *PDI *in the regulation of proinflammatory cytokine *TNF-α*, RAW 264.7 cells were transfected with *PDI *siRNA for 48 hours and *TNF-α *release into the cell supernatant was assessed. Transfection with 100 nM *PDI *siRNA produced an average 36.8% inhibition of the *PDI *gene expression (Figure 7a, *P *< 0.001). Interestingly, the *PDI *downregulation by siRNA caused a 3.19-fold increase in TNF-α release (Figure 7b, *P *< 0.001).

## Discussion

The notion that reduced/denatured proteins would spontaneously reoxidize and refold to form their native conformation led to the search for a physiological catalyst of this process. An enzyme was found that catalyzed the formation of native proteins from the reduced/denatured state and has been termed as *PDI *[38]. *PDI *is widely distributed and has been detected in most vertebrate tissues, although detailed studies have been confined to the enzyme from the liver. In the mammalian liver homogenates, *PDI *is found in crude microsomal membrane fractions [39]. In the rat liver, the enzyme co-sediments with markers of the endoplasmic reticulum [38]. *PDI *is a membrane-associated enzyme of the endoplasmic reticulum and its function, in part, is translational modification of proteins [40]. *PDI *may also catalyze the covalent crosslinking of native proteins or the covalent immobilization of biologically active molecules to the extracellular matrix.

In the present study, by using animal models of sepsis or endotoxemia, we have shown that the *PDI *gene expression is decreased at 20 hours after CLP or LPS infusion. Similarly, *PDI *gene expression is downregulated in a macrophage-like cell line after stimulation by LPS for 4 hours. These results indicate that *PDI *gene expression is downregulated under inflammatory conditions and that LPS plays an important role in the downregulation of *PDI*. In addition, to evaluate the role of *PDI *on *TNF-α *gene expression, we have used bacitracin, a specific inhibitor of *PDI*, on the *TNF-α *release and the expression in 24-hour-cultured RAW 264.7 cells. *TNF-α *levels in the supernatant and cellular *TNF-α *in RAW 264.7 cells cultured with bacitracin were significantly increased. In addition, we further confirmed that downregulation of *PDI *using *PDI *siRNA significantly increased *TNF-α *release from cells. These results suggest that *PDI *plays an important role in the production of proinflammatory cytokine *TNF-α*.

*PDI *has been found to be secreted from a variety of cell types, including hepatocytes [41], pancreatic exocrine cells [42], endothelial cells [43], and activated platelets [44]. While the biological importance of these secreted proteins remains in most cases obscure, the function of *PDI *secreted by thyrocytes into the lumen of the thyroid follicles has been identified [45]. It has been shown that the enzyme is involved in the control of thyroglobulin folding and multimerization, probably by reducing the intermolecular disulfide bridges and thus limiting the extent of multimer formation. While the full biological importance of the protein disulfide activity must still be understood, some interesting examples of *PDI *in pathological events such as Sindbis virus [46] and HIV [47] have been demonstrated. It has been suggested that *PDI *is specifically upregulated in response to hypoxia/ischemia in astrocytes [48]. In addition, the overexpression of this gene into neurons protects against apoptopic cell death induced by hypoxia/brain ischemia. Further studies by the same group indicate that ubiquilin, an endoplasmic reticulum-associated protein, together with *PDI*, has critical functions as a regulatory protein for cell death and therefore that upregulation of these proteins may result in the acquisition of tolerance against ischemic stress in glial cells [48]. A recent report also indicates that the transcriptional activity of *NF-κB *is negatively regulated by *PDI *[32]. Overexpression of *PDI *in RAW 264.7 cells strongly suppressed the LPS-induced production of inflammatory cytokines as well as *NF-κB*-dependent luciferase activity. This negative regulation of *NF-κB *was reversed by bacitracin, a *PDI *inhibitor. Finally, *PDI *expression was induced by the anti-inflammatory cytokine *IL-10*, and *IL-10*-mediated inhibition of LPS-induced *IL-6 *expression was reduced by bacitracin. These findings clearly demonstrate that *PDI *is a negative regulator of *NF-κB *and may act downstream of *IL-10 *in this signal pathway [32].

Our present study with septic rats, in which immunomodulation is known, also indicates that *PDI *is a regulator of inflammatory cytokines. Previous studies have demonstrated that proinflammatory cytokines play a critical role in the initiation and progression of sepsis syndrome and that *TNF-α*, *IL-1β*, and *IL-6 *are important mediators of hemodynamic, metabolic, and immunologic alterations in the host during sepsis [27-31]. Studies have also shown that circulating concentrations of *TNF-α*, *IL-1β*, and *IL-6 *increase significantly in the early, hyperdynamic stage of sepsis and remain elevated in the late, hypodynamic stage of sepsis [27,49]. In the present study, we have provided a clue that *TNF-α *release increased significantly in RAW 264.7 cells treated with bacitracin, which is an inhibitor of *PDI*. This result indicates the important role of *PDI *in *TNF-α *release in sepsis.

## Conclusion

In summary, our results indicate that *PDI *gene expression is downregulated in sepsis or endotoxemia. In addition, *PDI *gene expression is attenuated in a macrophage-like cell line after stimulation with LPS. Since the *PDI *inhibitor bacitracin significantly increases *TNF-α *release in a macrophage cell line, it appears that prevention of *PDI *downregulation may be a novel approach to reduce proinflammatory cytokine release in sepsis. Further studies are necessary in this direction.

## Key messages

• Protein disulfide isomerase (*PDI*), an important factor for the protein modification step in the post-translational event, plays an essential role in cell survival under stress conditions.

• In an experimental model, *PDI *gene and protein expressions were significantly downregulated in late sepsis.

• Similar downregulation was also observed in lipopolysaccharide-treated RAW 264.7 cells, a macrophage-like cell line.

• Bacitracin, a specific *PDI *inhibitor, significantly increased tumor necrosis factor-alpha (*TNF-α*) gene expression and *TNF-α *release as well as its cellular levels in a dose-dependent manner.

• Collectively, the data suggest that prevention of downregulation of *PDI *in sepsis attenuates hyperinflammation and reduces tissue injury.

## Abbreviations

BW = body weight; CLP = cecal ligation and puncture; ELISA = enzyme-linked immunosorbent assay; G3PDH = glyceraldehyde 3-phosphate dehydrogenase; IL = interleukin; LPS = lipopolysaccharide; NF-κB = nuclear factor-kappa-B; PCR = polymerase chain reaction; PDI = protein disulfide isomerase; RAW 264.7 = murine macrophage-like cell line; RT-PCR = reverse transcription-polymerase chain reaction; siRNA = short interfering RNA; TNF-α = tumor necrosis factor-alpha.

## Competing interests

The authors declare that they have no competing interests.

## Authors' contributions

MZ designed the study, collected data, interpreted the data, performed statistical analysis, and drafted the manuscript. NH is a summer student who helped MZ to collect the data. MM and RW participated in the design of the study. SRM and AJ participated in the critical revision of the manuscript. PW conceived of the study, participated in its design and interpretation, and helped to draft the manuscript. All authors read and approved the final manuscript.
